# Supplementary material for: Oral Administration of Probiotic Bifidobacterium breve Ameliorates Tonic–Clonic Seizure in a Pentylenetetrazole-Induced Kindling Mouse Model via Integrin-Linked Kinase Signaling
Source: Int J Mol Sci. 2024 Aug 27;25(17):9259. doi: 10.3390/ijms25179259 (PMC11395544; doi:10.3390/ijms25179259)
Supplement: Supplementary file 1 [file ijms-25-09259-s001.zip › ijms-3163895-supplementary.pdf]

Supplementary Material

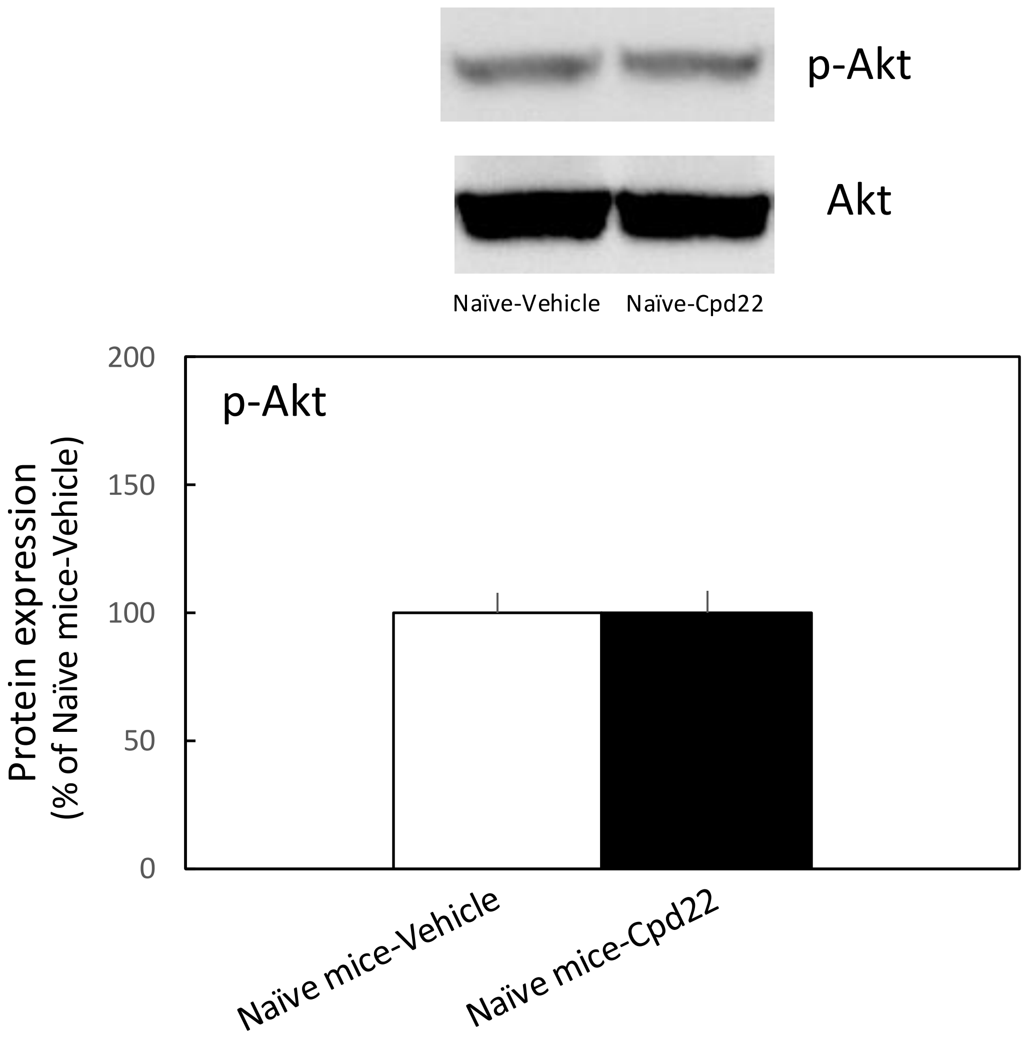

Figure S1. Effect of the ILK inhibitor Cpd22 on the basal expression level of p-Akt in the hippocampus of naïve mice. Cpd22 (10 mg/kg) or the vehicle was administrated intraperitoneally to naïve mice, and the brain was dissected 5 h after Cpd22 injection. The images show representative results (upper: p-Akt, lower: Akt). The p-Akt protein levels were normalized to Akt as a loading control. Results are shown as a percentage of protein expression levels in vehicle-treated naïve mice. Data are expressed as the mean ± SD: $n = 6$ per group. No significant differences were observed between groups (Student's *t*-test).

Table S1. Summary of mRNA and protein expression levels.

		CS	CB	PS(4h)	PB(4h)	PS(24h)	PB(24h)
**ILK**	mRNA		↑ Vs. CS	N.S.	N.S.	N.S.	N.S.
	Protein		↑ Vs. CS	N.S.	N.S.	N.S.	N.S.
**Neuropsin**	mRNA		↑ Vs. CS	↓ Vs. CS	N.S.	N.S.	N.S.
	Protein		N.S.	↓ Vs. CS	N.S.	N.S.	N.S.
**MMP-9**	mRNA		N.S.	N.S.	N.S.	N.S.	N.S.
				⇡ Increasing tendency		↓ Vs. PS(4h)+PB(4h)	
	Protein		N.S.	N.S.	N.S.	N.S.	↑ Vs. CS
**Iba1**	mRNA		N.S.	N.S.	N.S.	N.S.	N.S.
**BDNF**	mRNA		N.S.	N.S.	N.S.	N.S.	N.S.

CS, Control-Saline; CB, Control-*B. breve* A1; PS(4h), PTZ-Saline 4h; PB(4h), PTZ-*B. breve* A1 4h; PS(24h), PTZ-Saline 24h; PB(24h), PTZ-*B. breve* A1 24h. N.S. : Not significant.
